# The Quality—Not Quantity—Of the Work

**Published:** 1919-10-25

**Authors:** 


### "THE CURED PATIENT."

### The Quality—not Quantity—of the Work.

Noah, so I discovered when a boy, was clothed in a dignity that varied according to the amount you paid for his ark. I had a neighbour, I remember, whose father was very wealthy and who could pay forty shillings for an ark and think nothing of it. In this case the patriarch could head a procession of fifty pairs of animals, many more than he ought to have crowded into his ship for the passage to Ararat. My own dear folk were very, very poor and my little two-shilling ark, which meant so much to them and to me, carried a small cargo of ten pairs of animals and a funny little bit of painted wood which, I was told, was a dove. A little friend of mine, still poorer, bought an ark for threepence from an open-air stall one October Fair. His Noah was only a five-pair-man, and his procession, unpainted, looked most insignificant unless the animals were put up in single file; then they looked better, and quite good enough for shooting at with a toy cannon, the ultimate and unhappy fate of all Noah's Arks. I think it was in those far-distant happy days that I was impressed with the dignity of a long procession; it was a token of pomp and affluence. Now that I am a man I have put away childish things, and my little Noah's Ark has changed into a great hospital. But I have not quite put away childish ideas, I am afraid, for I still cling to my old love of the long procession. "Two hundred thousand out-patients were seen"; "Twenty thousand in-patients occupied our beds." There it is, you see. It is down in the annual report. I simply could not resist that. But I wonder whether I am right. After all, a hospital is not exactly an ark. The business does not end, as it did in the ark, when you get safely inside. When I tell you of that two hundred thousand, who walked in at one door and out at another, "seen," of course, as they passed, I wonder whether I am really telling you anything at all of work done (except by the clerk who counted them), but only of opportunities of doing work; am I not drawing attention to our responsibilities rather than to our merits? Am I not advertising our liabilities rather than our assets?

But if I give up my loved procession what have I left? How else can I tell you what a very fine fellow I am?

And I know that others, too, other hospital men, are in the same difficulty; men who entered hospital life about the same time as I did—how many years ago? Well, never mind. At that time we cried aloud from the depths, for we were deep in debt and deep in despair. We do not do that now. The public are tired of unbusinesslike habits and of pulling us out of quagmires. We now stand at the saluting-post and see the procession go by. But the procession is getting a little straggly and wobbly and unconvincing.

Let us face it, however it looks in the report. For this is the fact, that we have to fix some new standard to measure the work of a hospital. Its procession is not its output. What is? I read a paper by an American doctor, written before the war, on the "Product of a Hospital." He set down that the cured patients were the only test of the efficiency of a hospital; the "cured patient" was the only product. But are there no other products? Is not the education of the medical student a product? Is not the training of a faithful nurse a product? Is not the experience gained by the physician and surgeon a product? Is not the research patiently carried out and afterwards published a product? Is not the tone of the hospital, which insists that bluffing is common and that honesty in thought and work is all that matters—is not the tone of a great hospital a product? Are not all of these products, just as truly as the cure of the patient? No; they are not. For, if you think of it, these are by-products which depend entirely upon the output of the product itself. If the motto of the hospital is *not* "Cure the patient," then the education of the student is wrong education and of no value; if the nurse is not trained under that motto her training is no training at all. The same motto must be the stimulus of the physician and the surgeon; the same must inspire research; and, obviously, the tone of the hospital is a mockery if it is not inspired by a passion for making sick men and women healthy and well again. That is what Dr. Codman thought, to whom I have referred. I agree. The cured patient is the only product. The rest are by-products which can only exist if *the* product is being turned out plentifully. More than that, if the hospital is not producing that for which it primarily exists, then its by-products are tainted and are a danger; if its product is not good, its by-products are a centre of putrefaction and infection in the land. See to it that the hospital's output is in accordance with the motto and you need not trouble about education, research, training, experience, high tone. They will follow.

Clearly, the procession passing through is no longer a measure of our efficiency. It never was, really. Good work is done in spite of the procession, not because of it. The crowd is in the way. They must be dealt with somewhere, but elsewhere; they block the way at the hospital. The number who sat round the Pool of Bethesda is of small account. The most important question is—"How many stepped in, and what happened to them after?" "What happened to them after?" That is the very heart of the matter. A "follow-through" department must be one of the new departments of the hospital. A department to which a discharged patient may come every month or two months or three months and have a chat with the physician or surgeon responsible for his cure. It is in this department the real result of hospital work may be measured. It is there the physician may learn to modify his methods. It is there the surgeon will learn the true value of his operation. I was present at such a department the other morning. A dozen former patients had received a postcard to remind them that it was their day. Notes of their treatment when they were patients were on the desk before the surgeon. They were quite a happy group, and were pleased with the interest taken in them. Two "gastric ulcers" were seen first. The old wound was examined. Questions were asked: "Have you any pain or any kind of discomfort at all?" "Do you eat anything you like?" "Can you do a good normal day's work?" A few words of encouragement followed, and a request to come and have a chat in three months' time. Then came an old cancer of tongue. There was no sign of recurrence, and the patient was cheered. "Don't hesitate to drop in any Friday morning if anything worries you." Then two "breasts." The axilla was clear. One found difficulty in raising the arm readily. This was at once looked into. Nothing to worry about; the old wound was "pulling." It would get quite well. Then a poor

(*Continued on page* 85.)

**"The Cured Patient"**—(*continued from page 84*).
fellow with inoperable carcinoma, only thirty years old. The surgeon and the patient knew all about it. They had a little quiet talk in the corner. I saw them shake hands, and I overheard the surgeon say, "No; I can find no further sign at all." And so on through all the cases. And why cannot our out-patient departments become real "follow-through" departments? They cannot under present conditions. But the conditions are crying aloud for alteration of some kind. What good is it doing anyone when you ask the physician to examine twenty to thirty new cases in an afternoon? You are simply breaking the heart of a good man. And what waste of time, when you remember that many of those patients will not attend again.

In the next annual report I propose to say little about the procession. It is not a thing to be proud of. I wish I could say something like this; dare I?: "We have been able to cure completely several people who would otherwise have been permanently ill or would have died. They are now well and good citizens. In curing these we have fortunately been able to study the course of the disease very thoroughly, and in comparing our observations with those of our sister hospitals they and we have been able to trace the causes of this scourge. Papers published, and which are now in the hands of all private practitioners have drawn attention to the necessity of very early treatment of a definite kind, and we hope that the disease will be soon entirely destroyed," etc, etc. What if that could be said, as it will one day be said, of cancer and many other horrible things?

I think such a Report would be much more interesting than information as to the length of a procession, even though it reach "from London to Land's End."

My poor little Noah's Ark is all broken to pieces. I will hand it to some little child. E. W. M.

